# Development of an educational cartoon to prevent worm infections in Chinese schoolchildren

**DOI:** 10.1186/2049-9957-2-29

**Published:** 2013-12-02

**Authors:** Franziska A Bieri, Li-Ping Yuan, Yue-Sheng Li, Yong-Kang He, Andrew Bedford, Robert S Li, Feng-Ying Guo, Sheng-Ming Li, Gail M Williams, Donald P McManus, Giovanna Raso, Darren J Gray

**Affiliations:** 1 QIMR Berghofer Medical Research Institute, Herston, Brisbane, Queensland, Australia; 2 The School of Population Health, The University of Queensland, Brisbane, Queensland, Australia; 3 Hunan Institute of Parasitic Diseases, WHO Collaborating Centre for Research and Control on Schistosomiasis in Lake Region, Yueyang, People’s Republic of China; 4 Andrew Bedford Creative Services, Brisbane, Australia; 5 Swiss Tropical and Public Health Institute, Basel, Switzerland; 6 Centre Suisse de Recherches Scientifiques en Côte d’Ivoire, Abidjan, Côte d’Ivoire

**Keywords:** Soil-transmitted helminths, Neglected tropical diseases, Health promotion, Video-based health education package, Development of educational cartoon, Integrated control, Formative research, Pilot testing, Sustainable control of soil-transmitted helminths

## Abstract

**Background:**

With more than two billion people infected worldwide, soil-transmitted helminths (STH) are the most widespread infections. To date, STH control efforts rely predominantly on recurrent mass drug administration (MDA), which does not prevent reinfection. Additional public health measures including novel health educational tools are required for more sustained integrated control of STH. We describe the development of an educational cartoon video (*The Magic Glasses*) targeting STH infections in Chinese schoolchildren and its pilot testing in China.

We applied an extensive community-based mixed methods approach involving input from the target group of 9–10 year old schoolchildren and key informants, such as teachers, doctors and parents, in order to identify potential STH infection risks in the study area and to formulate key messages for the cartoon. The development of the educational cartoon included three major steps: formative research, production, and pilot testing and revision.

**Results:**

We found that most adults and approximately 50% of the schoolchildren were aware of roundworm (*Ascaris*) infection, but knowledge of transmission, prevention and treatment of STH was poor. Observations in the study area showed that unhygienic food practices, such as eating raw and unwashed fruit or playing in vegetable gardens previously fertilised with human faeces, posed major STH infection risks.

**Conclusions:**

It was crucial to assess the intellectual, emotional, social and cultural background of the target population prior to video production in order to integrate the key messages of the cartoon into everyday situations. Overall, our strategy for the development of the cartoon and its incorporation into a health education package proved successful, and we provide a summary of recommendations for the development of future educational videos based on our experiences in China.

## Multilingual abstract

Please see Additional file 1 for translation of the abstract into the six working languages of the United Nations.

## Background

Globally, more than two billion people are infected with soil-transmitted helminths (STHs), mainly in the developing nations of Asia, Africa and Latin America [1]. In China, STHs still impact substantially on public health with an estimated 129 million people infected. Children aged 5–14 years have the highest rates of infection. Major endemic foci are observed in the central, western and southern provinces [2].

Soil-transmitted helminths are intimately associated with rural poverty, inadequate sanitation and waste disposal, lack of clean water and poor hygiene, as well as limited access to health care and preventive measures through health education. These worms comprise the most common of the 17 major neglected tropical diseases (NTDs) which cause disabling chronic infections globally [3]. Most at risk are children and pregnant women in developing countries, where these infections are so common that children are most likely to harbour at least one, if not several, worm species [1]. It has been shown that STHs have a negative impact on physical, intellectual and cognitive development [4]. To date, control efforts rely predominantly on recurrent mass drug administration (MDA) with albendazole or mebendazole, but these do not prevent reinfection. It has been shown repeatedly that prevalence returns to the pre-treatment levels within 6–18 months after treatment cessation [3,5-7]. Additional public health measures, such as health education, are required to augment MDA for sustained integrated control of these intestinal worms leading to their elimination.

Our recent article published in the *New England Journal of Medicine* reported success in preventing STH infections in Chinese schoolchildren through a health education package incorporating a cartoon video [8]. Our study showed a 50% efficacy in preventing the incidence of STH infection and established proof of principle that the video-based health educational package increases student knowledge and improves hygiene practice, resulting in fewer worm infections. The video used in this study [1] was developed and produced by our multi-disciplinary, international team and is a 12-minute animated narrative cartoon entitled *The Magic Glasses*.

Here we describe the formative research conducted for the development of the cartoon, the production process and its pilot testing in China prior to its application in the subsequent published intervention trial [7].

## Methods

### Development and production of the educational cartoon

**Figure 1:**
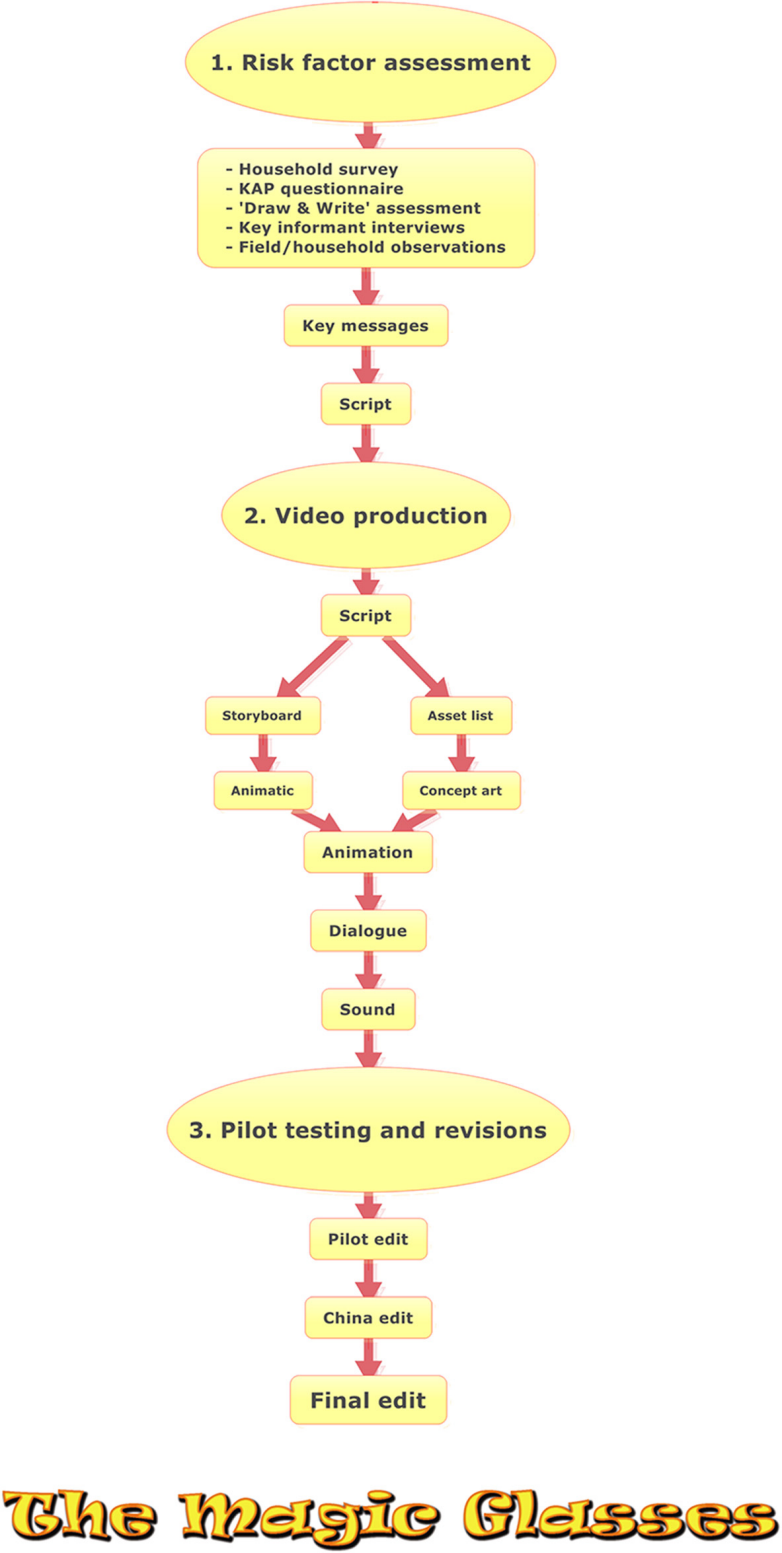
**Flow diagram showing the steps involved in the development of *The Magic Glasses *** **cartoon.**

**Figure 2:**
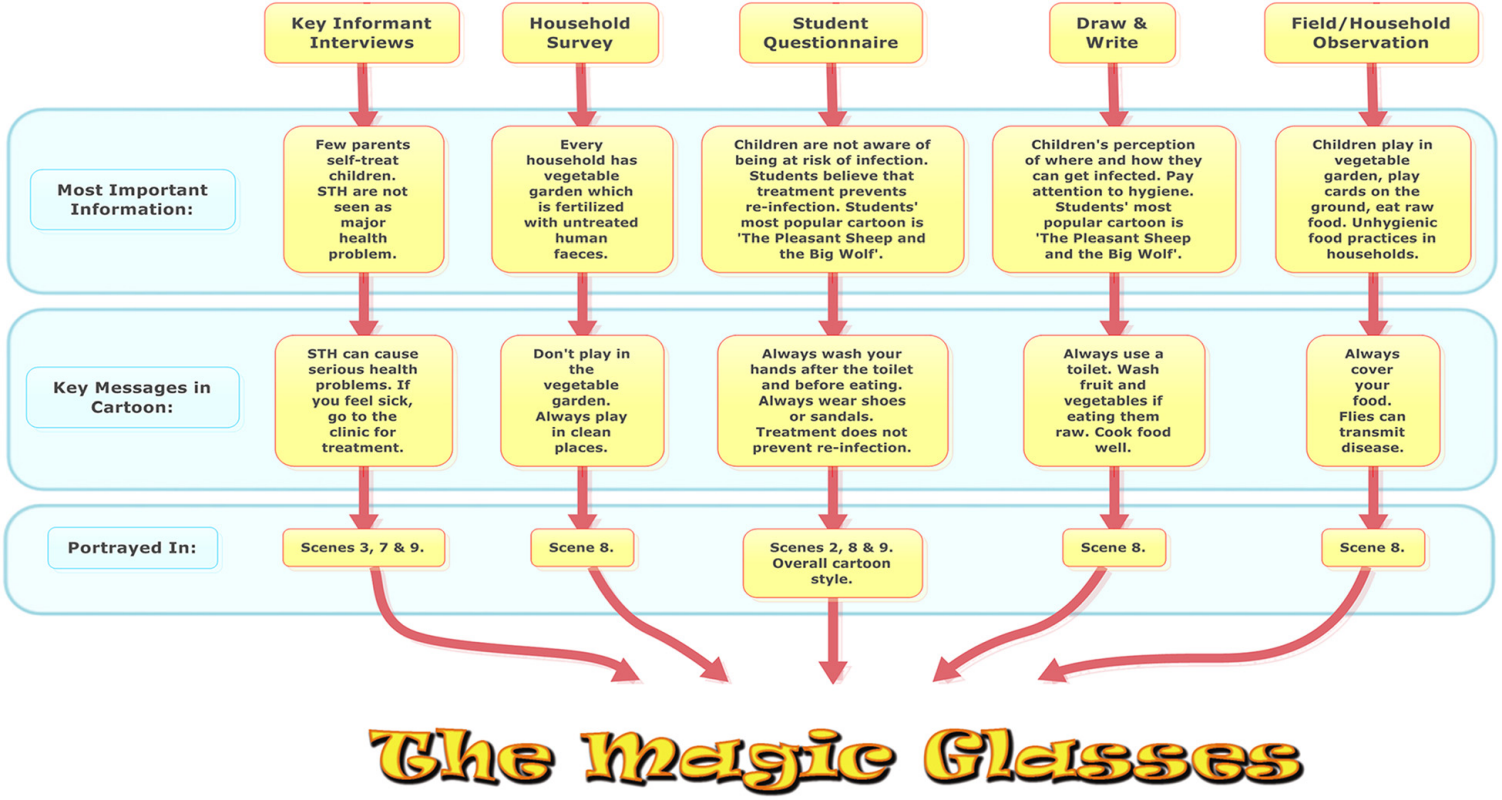
From defining risk factors to the key messages presented in the cartoon.

The development of the educational cartoon included three major steps: formative research, production, and pilot testing and revision (see Figure 1). Recommendations made by previous video-based studies, behavioural theories, didactic principles and teaching experiences were taken into consideration. Furthermore, research was conducted on Chinese animation history and favourite Chinese cartoons to choose a cartoon style popular among the target group (see Figure 2).

### Formative research

Formative research was conducted in Linxiang City District, China, in October 2009 to inform the cartoon development and production. This comprised of an assessment of the local background such as demographics, living conditions, previous knowledge about worms, hygiene practices, attitudes towards worms and infection risk factors for STHs. We used the following definition for ‘risk factor’: ‘An aspect of personal behaviour or lifestyle, or an environmental exposure, which is associated with health-related conditions considered important to prevent disease’ [9].

We applied an extensive community-based mixed methods approach involving input from the 9–10 year old student target group, and key informants such as teachers, doctors and parents in order to identify potential worm infection risks in the study area and to formulate key messages for the cartoon. The schoolchildren’s previous knowledge, attitudes and practice regarding STHs were assessed in quantitative questionnaires (N = 407), a qualitative drawing assessment and during in-depth interviews (N = 36).

**Figure 3:**
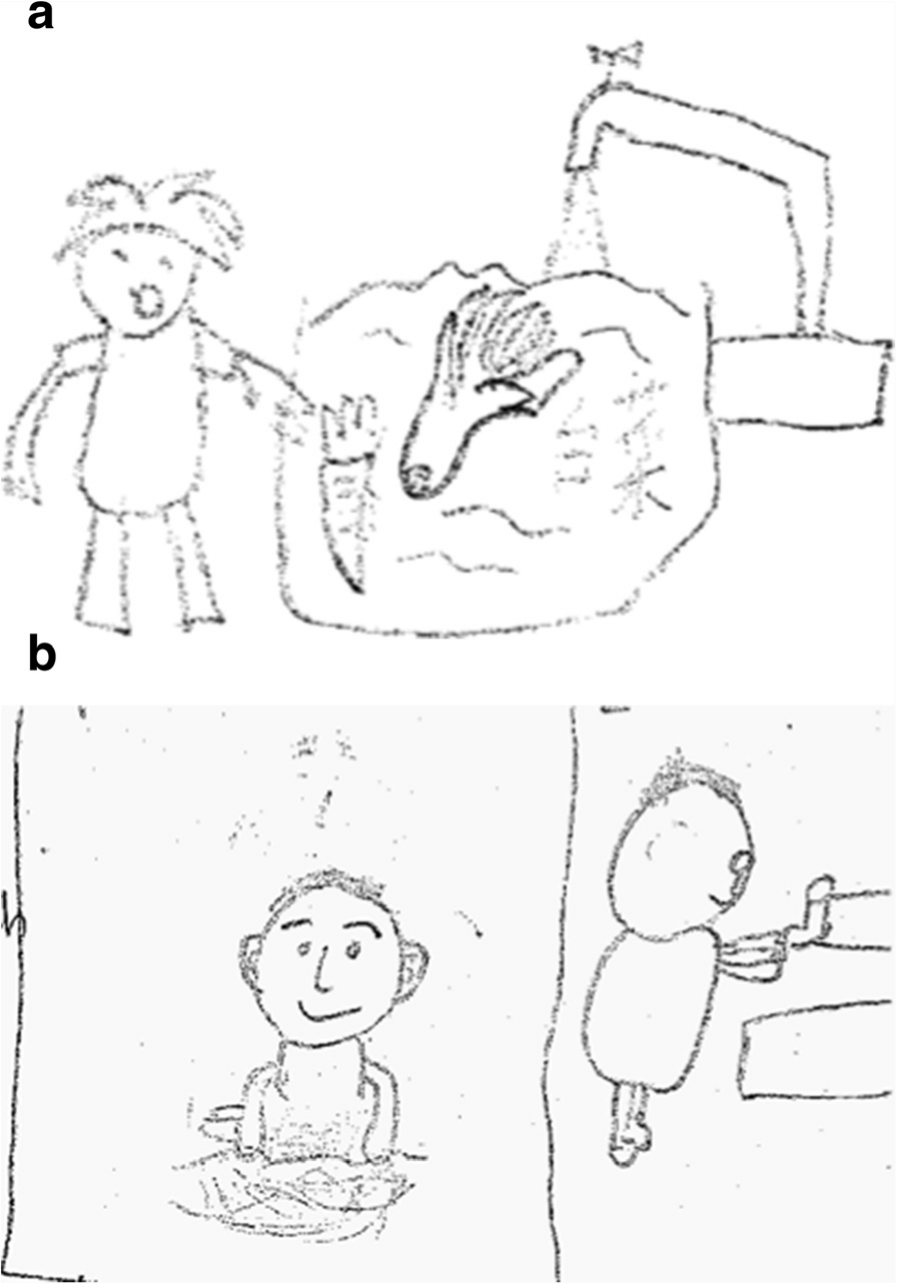
**Examples of drawing assessments. a)** Hygiene awareness: wash fruit and vegetables. **b)** Wash hands after playing in dirty places.

Risk factors reflecting the natural and human environment such as worm transmission hot spots, behaviour conducive to infection, and the intellectual, emotional, social and cultural background of the target student population were assessed in questionnaires, interviews, focus group discussions (FGDs), ‘draw and write’ assessments (see Figure 3), and during household and field observations. The different qualitative and quantitative approaches were triangulated in order to assess one set of information from various angles and with differing methodologies [10]. To avoid researcher bias, the assessments were standardised and conducted by both Chinese and Western researchers (researcher triangulation) [10]. The formative research involved the following:

•*A household survey* that included household observations, infrastructure assessment and in-depth interviews with the head of the household (N = 10, age range: 32–75 years, grandparents in seven households).

•*A knowledge, attitude and practice (KAP) questionnaire* with grade 4 schoolchildren (N = 407; 9–10 year-old). This consisted of: multiple choice questions relating to demographics; medical history; favourite comics and cartoons; previous health education and knowledge of STHs, their transmission, symptoms and treatment; attitude towards intestinal worm infections; and self-reported hygiene practice such as hand washing, food handling, toilet use and the wearing of shoes.

•*Qualitative ‘draw and write’ assessment and semi-structured interviews* assessing the schoolchildren’s previous knowledge of intestinal worms (N = 36; aged 9–10 years) (see Figure 3).

•*Key informant interviews* (KIIs) with teachers (one head of school, one teacher of mathematics), parents, a paediatrician, a health officer at Linxiang Centre for Disease Control (CDC) and an education officer at Linxiang City District Health Bureau.

•*Behaviour observations* to record risk behaviour/hygiene practices on video to identify risk factors for STH infection.

Observations in the field were recorded in writing and, where possible, on video camera. For the household surveys, a systematic infrastructure assessment was carried out using a standard observation form for each household.

### Production of The Magic Glasses cartoon

The cartoon was produced during the period of November 2009 to June 2010. In order to translate the assessed risk factors for acquiring intestinal worm infections into effective educational messages that stimulate schoolchildren to change their behaviour, behavioural theories including the Social Cognitive Theory [11,12], the Health Belief Model [12], the Transtheoretical Model [12] and Cognitive Theory of Multimedia Learning [13] were consulted. The key messages were then presented to a multi-disciplinary team of epidemiologists, education experts, animators and a scriptwriter. Over the course of several brainstorming sessions, a script for the cartoon narrative was drafted. During the scriptwriting process, Chinese scientists were consulted repeatedly for advice on China-specific cultural aspects. The script was a written document describing the dialogue, settings and characters from which all other elements essential for cartoon development were created. These included a storyboard to visualise camera shots and an animatic, turning the storyboard into a slideshow to pace and time the cartoon. Subsequently, concept artwork was created for all the main features presented in the script including the cartoon characters, the settings and general cartoon style. Next, resources were pooled together under the supervision of the cartoon director, and each stage was continually reviewed, iterated and placed into the movie. Backgrounds were created alongside characters, which were animated scene by scene using Adobe Creative Suite, Autodesk 3DS Max and MotionBuilder software. Dialogue and sound were then added. Throughout the process, results were discussed with the multi-disciplinary team and content was adapted accordingly. Figure 1 illustrates the different production steps in the development of the cartoon produced by a professional audiovisual company based in Brisbane, Australia. The audio was initially recorded in Australia with Brisbane-based Chinese film school students.

### Pilot testing

A pilot version of *The Magic Glasses* was tested in June 2010 in Linxiang City District, China. The cartoon was shown twice to an audience of schoolchildren (N = 80), teachers (N = 11) and invited parents (N = 9) in six schools prior to its use in the main intervention trial [1]. A short questionnaire was filled out by the audience during the second viewing to assess whether key messages in the video were understood. The answers were discussed in small FGDs with the schoolchildren, teachers and parents, and the audience was also asked to comment on the cartoon and make suggestions for improvement. As a result, the audio was rerecorded using professional voice actors based in China, which considerably improved the quality and entertainment value of the cartoon.

### Analysis

A Microsoft Access (Redmond, WA) database was used for data management [14]. SAS (SAS Institute, Cary, NC) and SPSS (IBM, Armonk, NY) software was used for statistical analysis.

#### Formative research

Frequencies of persons providing correct answers in the KAP questionnaire and household surveys were expressed as percentages. Interviews following the ‘draw and write’ assessment and KIIs were translated simultaneously and notes were taken in both Chinese and English by two investigators. The interviews were also recorded with the consent of the participants, transcribed into Chinese and later translated into English for further verification. Notes and transcripts were compared and analysed for content. Open-ended questions of the KAP questionnaire and the ‘draw and write’ assessment were subject to quantitative content analysis where all the answers were read and scanned for content and a code list created. Answers with similar meaning were categorised according to the code list, and frequencies for each of these answer categories were calculated and expressed as percentages.

#### Pilot testing

FGDs were translated simultaneously and notes were taken in both Chinese and English by two investigators. The questionnaire and focus group answers were entered in SPSS and frequencies of persons providing correct answers were expressed as percentages.

#### Ethics

Written ethical approval for the study was obtained from the human ethics committees of the Queensland Institute of Medical Research, Australia, and the Hunan Institute of Parasitic Diseases, China. Prior to commencement of the intervention, written informed consent was obtained from the parents or legal guardians of all student participants.

## Results

The results of the formative research including risk factor assessment are structured according to thematic groups of importance for the development of the cartoon.

### Formative research

#### Knowledge

Of the STHs, *Ascaris* was the most well known with 90% (95% CI (67–100) of adults and 51% (CI = 47–56) of schoolchildren, and all health and medical staff having heard of this parasite. Regarding hookworm, 50% (CI = 12–88) of adults and 11% (CI = 8.6–14.2) of schoolchildren had some knowledge, but *Trichuris* was unknown to adults, schoolchildren and key informants*.*

According to the student questionnaire, more than half of the schoolchildren did not know how they could get infected with intestinal worms (86%; CI = 75–98). A minority knew that infection could lead to abdominal pain (8%; CI = 0–17) and fatigue (3%; CI = 0–8). The majority (84%; CI = 82–100) knew they should pay attention to hygiene such as washing hands, washing fruit and vegetables, cooking food well and boiling water. Many schoolchildren (83%; CI = 71–96) thought they could acquire worm infections from worms on unwashed fruit/vegetable such as apples or by playing in water and grassland. This finding was confirmed in the ‘draw and write’ assessment (see Figure 3). In a few cases, STH infections were mistaken for schistosomiasis, of which the transmission (through water contact) was better known. Thirty percent (CI = 25–34) of the parents/grandparents, who were aware of STHs, self-treated their children/grandchildren. All the parents included in the household survey (N = 10) and 38% (CI = 33–42) (N = 407) of schoolchildren erroneously thought treatment also prevented reinfection; 48% (CI = 43–52) of the schoolchildren indicated that they did not know the answer to this question.

### Attitude

The KIIs revealed that no health officer, doctor, parent or student considered a STH infection to be a major health problem in the area where the most commonly perceived diseases were cardiovascular problems, influenza, hepatitis B and myopia. More than half of the schoolchildren (56%; CI = 52–61) did not think they were at risk of contracting STHs. Schoolchildren clearly associated intestinal worm infections with poor hygiene, as typified by the following statement: ‘If we pay attention to personal hygiene, it is not that easy for worms to enter our body.’ In the community, hygiene was mainly associated with safe food handling, access to safe drinking water and washing hands, but also included vague perceptions such as: ‘When we clean the floor, the dust will enter our body, which will lead to an infection.’ The knowledge and perception of STHs by parents and grandparents had a considerable impact on the attitude of the schoolchildren – as one student commented: ‘Mum told me I’m infected if I have stomach ache.’

The majority of schoolchildren (74%; CI = 70–78) indicated they would be anxious if they had an intestinal worm infection. One third (30%; CI = 25–34) did not know why they were anxious, but some schoolchildren mentioned that they were afraid of contracting disease caused by intestinal worms because it could not be cured (7%; CI = 4–9), would cause (abdominal) pain and diarrhoea (9%; CI = 6–12), or even death (3%; CI = 1–4). Schoolchildren also believed that drug administration could prevent reinfection: ‘I got this disease when I was young and I then took medicine, so I won’t have it anymore.’

### Health education and preferred media

According to the in-depth interviews with the teachers, health education was part of the curriculum in grades 3 and 4 for one to two weekly lessons on the subject ‘Life and Health’. However, STH infections were not addressed in these lessons and the teachers’ knowledge of intestinal parasites was generally poor (they did not know about worm transmission and prevention).

The schoolchildren’s favourite cartoons were episodes of *Pleasant Goat and Big, Big Wolf*, a funny, colourful and fast-paced Chinese animated television series. The schoolchildren liked this programme because it was funny (14%; CI = 10–17), interesting (12%; CI = 5–15) and the main character was clever (5%; CI = 3–7); 26% (CI = 22–31) did not indicate why they liked it or did not answer the question (43%; CI = 38–47).

### Important household and field observations

Behaviour conducive to STH infection was observed repeatedly in the study area and scenes reflecting the associated risks were integrated into the cartoon. One of the children’s favoured games was playing cards on the floor or ground which, if contaminated with worm eggs or larvae, posed a potentially serious infection risk. Children also played in vegetable gardens previously fertilised with human faeces. They had close contact with soil and often ate raw and unwashed root vegetables such as radishes and sweet potatoes. This reflected the earlier described perception of both adults and children that the most likely source of infection was eating raw and contaminated food. Another likely transmission pathway noted occurred through flies transporting intestinal worm eggs onto uncovered food.

Sanitation in the schools and households was poor with a lack of hand-washing facilities and latrines comprising open manure pits. Soap was available in households, but in none of the schools visited. Most living room (93%; CI = 91–95) and bathroom floors (89%; CI = 86–91) were either made of cement, porcelain or wood with only a few houses having unsealed floors. In the schools, the floors of toilets and classrooms were made of concrete, but the schoolyard was usually unsealed.

### Key messages for the cartoon

The formative research and risk factor assessment led to the formulation of the following key messages:

•Always play in clean places;

•Avoid playing in vegetable gardens;

•Always use a toilet;

•Always wash your hands after the toilet and before eating;

•Always cover food;

•Wash fruit and vegetables if eating them raw;

•Cook food well;

•Always wear shoes or sandals; and

•If you feel sick, go to the clinic for treatment.

**Figure 4:**
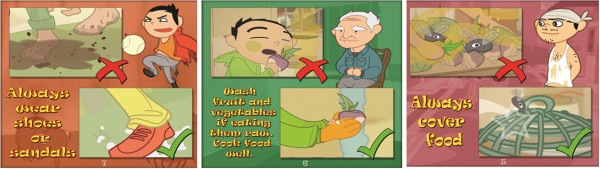
**Pamphlet with a selection of key messages (see Additional files**2**: Figure S1 for the entire pamphlet).**

These key messages were incorporated into the various scenes of the cartoon, reflecting the environment of the target group in Linxiang City District. This process is illustrated in a flow diagram (see Figure 2). The key messages were also compiled into a complementary pamphlet that was distributed during the course of the intervention (see Figure 4 and Additional file 2: Figure S1). Overall, the cartoon displays correct behaviour and creates confidence that improvement in hygiene practice results in a positive health outcome [12,15]. The storyline is summarised below and the video can be accessed online at:

http://www.qimr.edu.au/page/Home/Magic_glasses/, and

http://www.nejm.org/doi/full/10.1056/NEJMoa1204885

### Storyline of *The Magic Glasses*

*The Magic Glasses* is a 12-minute colour animated cartoon in the form of an entertaining narrative that informs about STH transmission and prevention. When nine-year-old Xiaoxiong puts on the ‘Magic Glasses’ given to him by a local doctor, he can see worm eggs and larvae in the village in bright yellow colours. Together with his friend, Xiaofang, he walks through the village and sees that many places and people are affected by the worms. He decides to educate the people about the worms and show them how to avoid an infection. Thanks to his efforts, the village is soon worm free, but Xiaoxiong warns everyone that they must keep up their good hygiene, otherwise the worms will come back.

The video was integrated into a health education package including classroom discussions, drawing and essay competitions, and a pamphlet containing relevant information on the transmission and prevention of STH infections. This allowed the schoolchildren to practice and consolidate their newly-acquired knowledge.

### Local control efforts for STH

According to health and education officials from Linxiang City District, a mass deworming programme had been conducted in the study area between 1994 and 2004. However, it had limited success due to a lack of communication between local authorities with schools and parents, resulting in poor treatment compliance.

### Pilot testing of the cartoon

The FGD and questionnaire results following the pilot testing of the cartoon showed that adults and schoolchildren readily understood the key messages, with questions about infection risk and symptoms for STHs being answered correctly by 82% (CI = 78–87) and 73% (CI = 67–79) of children, respectively. The feedback on the cartoon was generally positive with one major criticism concerning the audio. The language and voices recorded in Brisbane with the Chinese film school students were too formal and did not match the general cartoon style. Therefore, with the help of young Chinese researchers familiar with the colloquial expressions used by schoolchildren, the dialogue was reworded and the audio rerecorded using professional voice actors based in China.

## Discussion

We describe the development and pilot testing of the cartoon video *The Magic Glasses* targeting intestinal worm prevention in Chinese schoolchildren, subsequently used as part of a successful health education package in an intervention trial in China [1]. Critical information for the cartoon development was assessed in a formative research phase, which revealed that most adults and approximately 50% of the schoolchildren were aware of *Ascaris* infection, but knowledge of transmission, prevention and treatment of STHs was poor. Only 37% of the children thought they were at risk of getting infected with worms. Among the parents/guardians included in the household survey, 30% self-treated their children and everyone believed that treatment also prevented reinfection. Unhygienic food practices such as eating raw and unwashed fruit or playing in vegetable gardens, previously fertilised with human faeces, were perceived as major risks for STH infection.

According to the Health Belief Model, the level of awareness (perceived susceptibility) for a disease is a strong predictor of preventive health behaviour [12]. Therefore, the lack of knowledge and awareness combined with poor hygiene and sanitation in Linxiang City District would likely lead to repeated infection with intestinal worms. This meant that alongside the key points informing about prevention, the educational cartoon needed to include the following key messages:

1) Raise awareness of STH among the target group. Message: ‘You are at risk of getting infected’ (perceived susceptibility) [15].

2) Convince the target group that it is in their hands to change behaviour and thereby decrease infection risk (self-efficacy). Message: ‘You can protect yourself against STH by improving your hygiene practice’ [12].

For this purpose it was absolutely crucial to assess the intellectual, emotional, social and cultural background of the target population, prior to cartoon production, in order to integrate the key messages of the cartoon into everyday situations that children could identify with. As highlighted by others [12,16,17], an individual’s assimilation of scientific knowledge alone does not necessarily result in behavioural change. Behaviour is related to perceptions, values, power relationships and feelings, and cannot be changed simply with the acquisition of knowledge.

Accordingly, the key messages coming out of the formative research were translated into a narrative by a team of epidemiologists, educators, parasitologists and animators, thereby allowing the development of a culturally-tailored educational package that was both informative and engaging [18-20]. Again, consulting behavioural models and didactic principles informed the need to bring the information across to induce behaviour change.

## Conclusion

Overall, our strategy (see Recommendations below) for the development of the cartoon *The Magic Glasses* and its incorporation into a health education package proved successful [8]. An educational video provides an ideal basis for health education at school since it can be readily disseminated and reused several times, which may substantially increase cost-effectiveness. A need for innovative and effective educational tools that can be integrated into existing control efforts for STH infections and other neglected tropical diseases has been expressed in ‘A Research Agenda for Helminth Disease’ published in *PLoS Neglected Tropical Diseases* in April 2012 [21]. Interventions including health education to prevent worm reinfection are urgently required to augment the sustainability and effectiveness of chemotherapy as part of an integrated approach. Furthermore, the video-based health educational tool presented here suitably complements the MDA approach advocated by the World Health Organization for the future control of intestinal worm infections.

### Recommendations for the development of an educational cartoon video based on our experience of producing *The Magic Glasses*

1) Involve the local community and the target group early on in the formative research phase including in risk factor and context assessments.

2) Undertake careful formative research including assessment of risk factors and context using multiple, both quantitative and qualitative, methods.

3) Translation of risk factors into educational key messages should be grounded in behavioural theories [12].

4) Ensure the video incorporates instructional messages into a real-life situation displaying correct behaviour embedded in the local context, rather than depicting a stand-alone instructional message. Ideally the educational material should be developed locally to account for cultural differences.

5) Ensure the video is produced professionally by hiring a professional audiovisual company. It is also essential to involve an experienced scriptwriter.

6) Ensure the knowledge can be integrated into an entertaining narrative, thereby informing and entertaining at the same time.

7) Pilot test the video in the targeted area, and include feedback from the local community and targeted group.

8) Ideally, combine the video with other teaching methods such as class discussions or role-plays, allowing children to practice, consolidate and repeat the newly-acquired knowledge.

We recommend combining the video with other teaching methods if used as part of a school-based intervention to reinforce and consolidate the educational message. Ideally, the wider community, especially parents, should be included in the intervention, as this increases household involvement and increased levels of prevention. To achieve this, parents could be invited to participate in an annual school health day, whereby the video is shown and information on worm infection and prevention is distributed. Another option to extend the educational intervention to a wider public would be to broadcast the video on television, where it could be integrated in a health promotion television series with each episode targeting a different topic of public health importance such as promoting a healthy diet or preventing road accidents. The television series could be reinforced regularly in order to repeat the message, which increases sustainability of the programme. Such long-term interventions have proven very effective in large-scale community health promotion trials applying a multi-disciplinary approach [22,23]. The health education package we have developed that prevents worm infections supports the message that close collaboration between experts of different disciplines is needed to develop successful public health interventions with the potential to contribute significantly to better global health.

## Competing interests

The authors declare that they have no competing interests.

## Authors’ contributions

FB carried out the literature review; designed the research project; conducted the fieldwork, data collection, analysis and interpretation; and wrote the first draft of the manuscript. DG, GW, GR, YL and DM were responsible for the conceptualisation and design. YL, LY, YH, FG and SL were responsible for the fieldwork and data collection. DG, GW and DM participated in data analysis and interpretation. DG, GW, YL, GR and DM helped write the manuscript. DG, GW, DM, YL, LY, RL, YH, FG and SL contributed to the video development. AB was responsible for the video production. RL was responsible for the database. All authors read and approved the final manuscript.

## Supplementary Material

Additional file 1 Multilingual abstracts in the six official working languages of the United Nations. Click here for file

Additional file 2 Pamphlet with the key messages of the cartoon as a PDF. Click here for file
