# Supplementary material for: Development of an educational cartoon to prevent worm infections in Chinese schoolchildren
Source: Infect Dis Poverty. 2013 Dec 2;2:29. doi: 10.1186/2049-9957-2-29 (PMC4177148; doi:10.1186/2049-9957-2-29)
Supplement: Additional file 1 — Multilingual abstracts in the six official working languages of the United Nations. [file 2049-9957-2-29-S1.pdf]

Translation of the abstract into the six official working languages of the United Nations

تطوير رسوم مُتحركة تعليمية للحد من الإصابة بعدوى الديدان في المدارس الصينية

فرانشيسكا أي بيري، لى بينج يوان، يوي شنج لى، يونج كانج هي، أندرو بيدفورد، روبرت إس لي، فنج يينج جوا، شنج مينج لي،جيل إم ويليامز، دونالد بي ماكمانوس، جيوفانا راسو، دارين جي جراي.

الملخص

الخلفية: مع أكثر من ملياري شخص مُصاب حول العالم، إن الديدان المنقولة بالتربة (STH) هي العدوى الأكثر إنتشاراً. وإلى الآن، تعتمد جهود مكافحة الديدان المنقولة بواسطة التربة (STH)، في الغالب على الاستخدام المتكرر للدواء الشامل (MDA) الذي لا يمنع الإصابة مرة أخرى. وعليه، يلزم إتخاذ تدابير الصحة العامة الإيضافية بما في ذلك الأدوات الصحية التي تتضمن قصص روائية التعليمية من أجل الحفاظ على استدامة أكثر ومكافحة متكاملة لتلك الديدان. ونحن هنا نصف تطوير فيديو الرسوم الكرتوني التعليمي (النظارة السحرية) الذي يستهدف موضوع الالتهابات الناتجة عن هذه الديدان في المدارس الصينية والاختبارات التجريبية في الصين.

الأساليب: لقد طبقنا أساليب مُتباينة على نطاق مُجتمعي واسع تناول نهج المدخلات من الفئة المُستهدفة من طلبة المدارس 9-10 عاماً والمعلومات الرئيسية، مثل المُعلمين، والأطباء، والآباء والأمهات، وذلك من أجل تحديد المخـاطر المُحتملة من عدوى تلك الديدان في منطقة الدراسـة ولصياغة الرسائل التوعوية الرئيسية للكارتون. وقد شمل تطوير الرسوم المتحركة التعليمية ثلاث خطوات رئيسية هي: البحوث التكوينية، والإنتاج، والاختبار التجريبي والمراجعة.

النتـائج: وجدنا أن معظم البالغين وحوالي 50 ٪ من تلاميذ المدارس كانوا على علم بعدوى دودة (الإسكارس)، ولكن معلوماتهم عن كيفية انتقالها أو الوقاية منها أو علاجها كانت شبه معدومة. وقد لاحظنا في منطقـة الدراسة أن المُمارسـات الغذائية غير الصحية، مثل تنـاول الفواكة النيئة أو الغير مغسولة أو اللعب في الحقول الزراعية المُسَمّدة سابقاً بالبراز البشري، تُشكل مخاطر العدوى الأساسية للديدان المنقولة بالتربة.

الاستنتاجات: وعليه كان من الأهمية بمكان تقييم الخلفية الفكرية والعاطفية والاجتماعية والثقافية للسُكان المُستهدفين قبل إنتاج الفيديو من أجل إدماج الرسائل الأساسية من الرسوم المتحركة في مواقف الحياة اليومية. وعموماً، فقد أثبتت استراتيجيتنا لتطوير الرسوم المتحركة وإدماجها في عروض التثقيف الصحي كانت ناجحة، ونحن نقدم موجزاً لتوصيات تطوير أشرطة الفيديو الكرتونية التعليمية في المستقبل على أساس تجاربتنا في الصين.

Translated from English version into Arabic by Freelanceabunar, through

TRANSLATORS
WITHOUT BORDERS

研发卡通教育片预防中国学龄儿童蠕虫感染

Franziska A. Bieri, Li-Ping Yuan, Yue-Sheng Li, Yong-Kang He, Andrew Bedford, Robert S. Li, Feng-Ying Guo, Sheng-Ming Li, Gail M. Williams, Donald P. McManus, Giovanna Raso, Darren J. Gray

摘要

引言：土源性蠕虫是全球最常见的感染，超过 20 亿人感染。目前，土源性蠕虫病的控制主要依赖于反复的大规模化疗，但其无法阻止重复感染。需要整合一些其他的公共卫生措施如创新的健康教育手段来防治土源性蠕虫感染，以促进可持续性。我们研发了针对中国学龄儿童土源性蠕虫感染防治的卡通教育片（“魔镜”），并对其进行了测试。

方法：我们采用了广泛的、基于社区的综合方法，涉及了 9-10 岁的目标学龄儿童，以及与其密切相关的人群，包括老师、医生和父母，以便发现研究区域土源性蠕虫感染的潜在危险因素，并将其转化成卡通片中的关键信息。该卡通教育片的研发包括 3 个步骤，即信息收集、制作以及试点和修改。

结果：我们发现绝大多数成人和大约一半的学龄儿童听说过蛔虫，但是他们对于土源性蠕虫的感染方式、预防和治疗知之甚少。通过观察发现，不卫生的饮食习惯如吃生的和未洗净的水果、在先前用粪便施肥的菜园里玩耍是主要的危险因素。

结论：在卡通教育片制作前，对目标人群的知识、情感、社会和文化背景进行分析至关重要，以便于将日程生活场景整合进卡通片的关键信息中。总体上，我们研发该卡通片的策略及将其整合进一套健康教育包中的措施是成功的。基于我们在中国的经验，我们总结了未来研发健康教育片的一些建议。

Translated from English version into Chinese by Qian Men-bao, through

CDC CHINESE CENTER FOR DISEASE CONTROL AND PREVENTION
NATIONAL INSTITUTE FOR PARASITIC DISEASES

Développement d'un dessin animé éducatif sur la prévention des helminthiases chez les écoliers chinois

Franziska A. Bieri, Li-Ping Yuan, Yue-Sheng Li, Yong-Kang He, Andrew Bedford, Robert S. Li, Feng-Ying Guo, Sheng-Ming Li, Gail M. Williams, Donald P. McManus, Giovanna Raso, Darren J. Gray

Résumé

Contexte : Avec plus de deux milliards de personnes infectées dans le monde, les infections par helminthes transmis par le sol (HTS) sont les infections les plus répandues. À ce jour, les efforts de lutte contre les HTS reposent principalement sur l'administration massive et récurrente de médicaments, ce qui n'empêche pas les réinfections. Des mesures supplémentaires de santé publique, en ce compris de nouveaux outils éducatifs sur la santé, sont nécessaires pour un contrôle intégré plus soutenu des HTS. Nous décrivons le développement d'un dessin animé éducatif (Les lunettes magiques) ciblant les infections par HTS chez les écoliers chinois et ses essais pilotes en Chine.

Méthodes : Nous avons appliqué une vaste approche communautaire par méthodes mixtes, impliquant la contribution du groupe cible d'écoliers de 9-10 ans et d'informateurs clés, tels que les enseignants, les médecins et les parents, afin d'identifier les risques potentiels d'infection par HTS dans la région de l'étude et de formuler des messages clés pour le dessin animé. Le développement du dessin animé comportait trois grandes étapes : la recherche formative, la production et l'essai pilote et la révision.

Résultats : Nous avons constaté que la plupart des adultes et environ 50 % des élèves étaient au courant de l'infection par ascaris, mais que leur connaissance sur la transmission, la prévention et le traitement des HTS était limitée. Les observations dans la région de l'étude ont montré que des pratiques alimentaires insalubres, comme la consommation de fruits crus et non lavés ou les jeux dans des jardins potagers fertilisés avec des excréments humains, représentent des risques importants d'infection par HTS.

Conclusions : Il était crucial d'évaluer le contexte intellectuel, affectif, social et culturel de la population cible avant la production de la vidéo, afin d'intégrer les messages clés du dessin animé dans des situations quotidiennes. Dans l'ensemble, notre stratégie de développement du dessin animé et de son incorporation dans un programme d'éducation à la santé a fait ses

preuves, et nous donnons un résumé des recommandations pour le développement de futures vidéos éducatives, sur la base de nos expériences en Chine.

Translated from English version into French by Jacek Sierakowski, through

TRANSLATORS
WITHOUT BORDERS

Создание поучительного мультфильма для профилактики гельминтоза среди китайских школьников

Франциска А. Бриери, Ли-Пин Юань, Юэ-Шэн Ли, Юн-Кан Хэ, Эндрю Бедфорд, Роберт. С. Ли, Фэн-Ин Го, Шэн-Мин Ли, Гейл М. Уильямс, Дональд П. МакМанус, Джованна Расо, Даррен Дж. Грей

Аннотация

Общая информация: Более двух миллионов людей по всему миру заражены геогельминты, что делает их самым распространенным паразитом на земле. Современные методы борьбы с глистной инвазией основаны преимущественно на регулярном применении лекарственной терапии, что не предотвращает повторное заражение. Более устойчивый и комплексный контроль заражения гельминтами требует дополнительных мер в области общественного здравоохранения, в том числе применения нетрадиционных инструментов санитарного просвещения. В данной работе описан процесс создания поучительного мультфильма под названием The Magic Glasses («Волшебные очки») на тему заражения геогельминтами среди китайских школьников и его пробного проката в Китае.

Методология: Мы применили расширенный подход с использованием различных методик и общественных исследований и опирались на результаты опроса целевых групп школьников 9–10 лет, а также ключевых опрашиваемых: педагогов, врачей и родителей, с целью выявления возможных рисков заражения геогельминтами в исследуемых регионах и формулирования ключевых идей мультфильма. Создание поучительного мультфильма включало три основных этапа: формативное исследование, производство и пробный показ с внесением корректив на основе полученных результатов.

Результаты: Мы выяснили, что большинство взрослых и примерно 50% детей школьного возраста знакомы с таким явлением, как аскаридоз (заражение круглыми глистами), однако их знания о механизмах передачи, способах профилактики и лечения гельминтозов недостаточны. Наблюдения в исследуемых регионах показали, что основным фактором заражения гельминтами является отсутствие гигиены питания,

например употребление сырых и немытых фруктов или игры в саду или огороде, который удобрялся человеческими фекалиями.

Выводы: Было крайне важно оценить интеллектуальные, эмоциональные, социальные и культурные особенности целевого населения до начала анимационного производства для того, чтобы ключевые идеи мультфильма согласовались с повседневными ситуациями. В целом наша стратегия создания мультфильма и его внедрения в кампанию санитарного просвещения оказалась успешной, поэтому мы предоставляем общие рекомендации по созданию будущих познавательно-поучительных видеороликов, опираясь на опыт, полученный нами в Китае.

Translated from English version into Russian by Cristina Rychkova, through

TRANSLATORS
WITHOUT BORDERS

Elaboración de un video educativo de dibujos animados para evitar infecciones por gusanos en escolares chinos

Franziska A. Bieri, Li-Ping Yuan, Yue-Sheng Li, Yong-Kang He, Andrew Bedford, Robert S. Li, Feng-Ying Guo, Sheng-Ming Li, Gail M. Williams, Donald P. McManus, Giovanna Raso, Darren J. Gray

Resumen

Antecedentes: Con más de dos mil millones de personas infectadas en todo el mundo, los helmintos transmitidos por el suelo (STH) representan las infecciones más extendidas. Hasta la fecha, los esfuerzos para controlar los STH se basan principalmente en la recurrente administración masiva de medicamentos (AMM), que no evita la reinfección. Para conseguir un control integrado más continuado de los STH se requieren medidas de salud pública adicionales, tales como nuevos instrumentos de educación sanitaria. Describimos la elaboración del video educativo de dibujos animados The Magic Glasses (Las gafas mágicas) centrado en las infecciones por STH en escolares chinos y la correspondiente prueba piloto en China.

Métodos: Hemos aplicado un enfoque exhaustivo con distintos métodos basados en la comunidad, que implica la obtención de datos de un grupo de escolares de 9 a 10 años y de informantes clave, tales como profesores, médicos y padres/madres, con el fin de identificar riesgos potenciales de infección por STH en el área de estudio para formular los mensajes clave del video. La elaboración del video educativo de dibujos animados incluye tres etapas principales: investigación formativa, producción y prueba piloto/revisión.

Resultados: Hemos descubierto que la mayoría de los adultos y aproximadamente el 50 % de los escolares sabían de la existencia de la infección por gusanos redondos (Ascaris), pero los conocimientos sobre la transmisión, la prevención y el tratamiento de STH era pobre. En el área de estudio se observó que las prácticas alimentarias no higiénicas, tales como ingerir fruta cruda y sin lavar o jugar en huertos previamente fertilizados con heces humanas implican un importante riesgo de infección por STH.

Conclusiones: Fue crucial evaluar el nivel intelectual, emocional, social y cultural del grupo de personas objeto del estudio antes de la elaboración del video para integrar los mensajes clave del mismo en las situaciones cotidianas. En términos generales, nuestra estrategia para

la elaboración del video y su incorporación en un paquete de educación sanitaria han resultado exitosas. Así mismo, hemos podido recoger un resumen de recomendaciones para el desarrollo de futuros videos educativos basados en nuestras experiencias en China.

Translated from English version into Spanish by Mnprado, through

TRANSLATORS
WITHOUT BORDERS
