# Supplementary figures and images for: Development of an educational cartoon to prevent worm infections in Chinese schoolchildren
Source: Infect Dis Poverty. 2013 Dec 2;2:29. doi: 10.1186/2049-9957-2-29 (PMC4177148; doi:10.1186/2049-9957-2-29)

**Additional File 1: Pamphlet with the key messages of the cartoon**


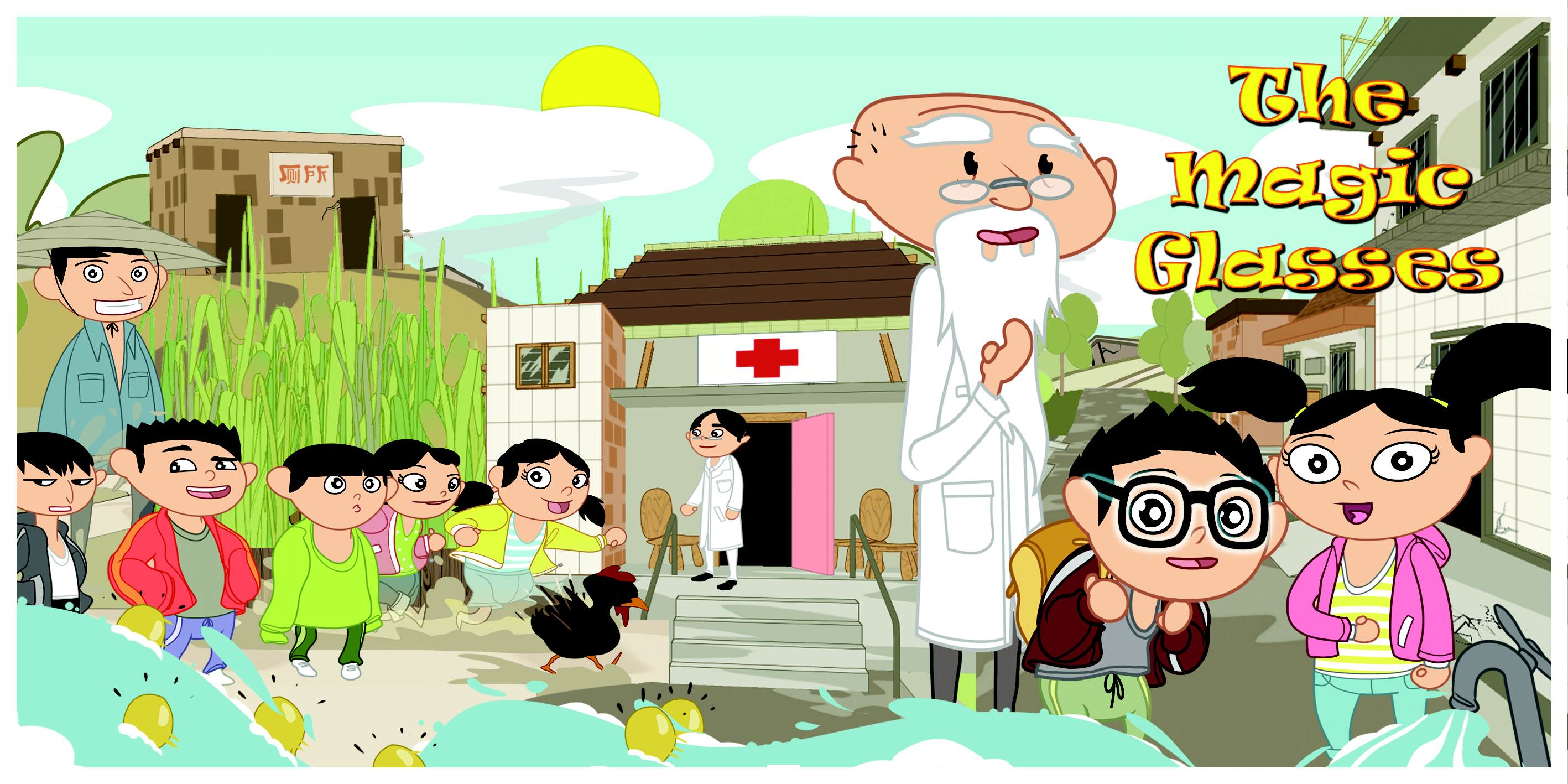

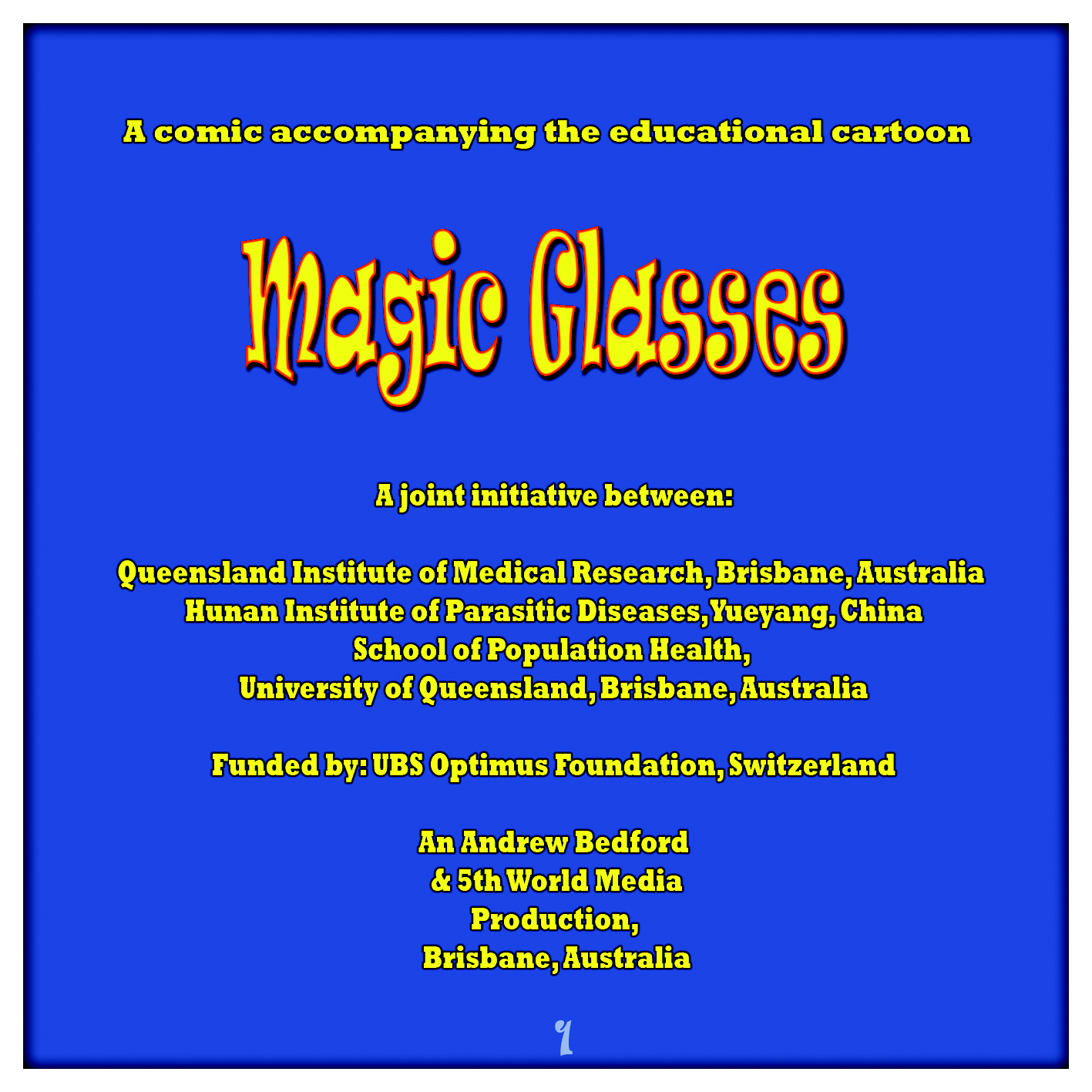

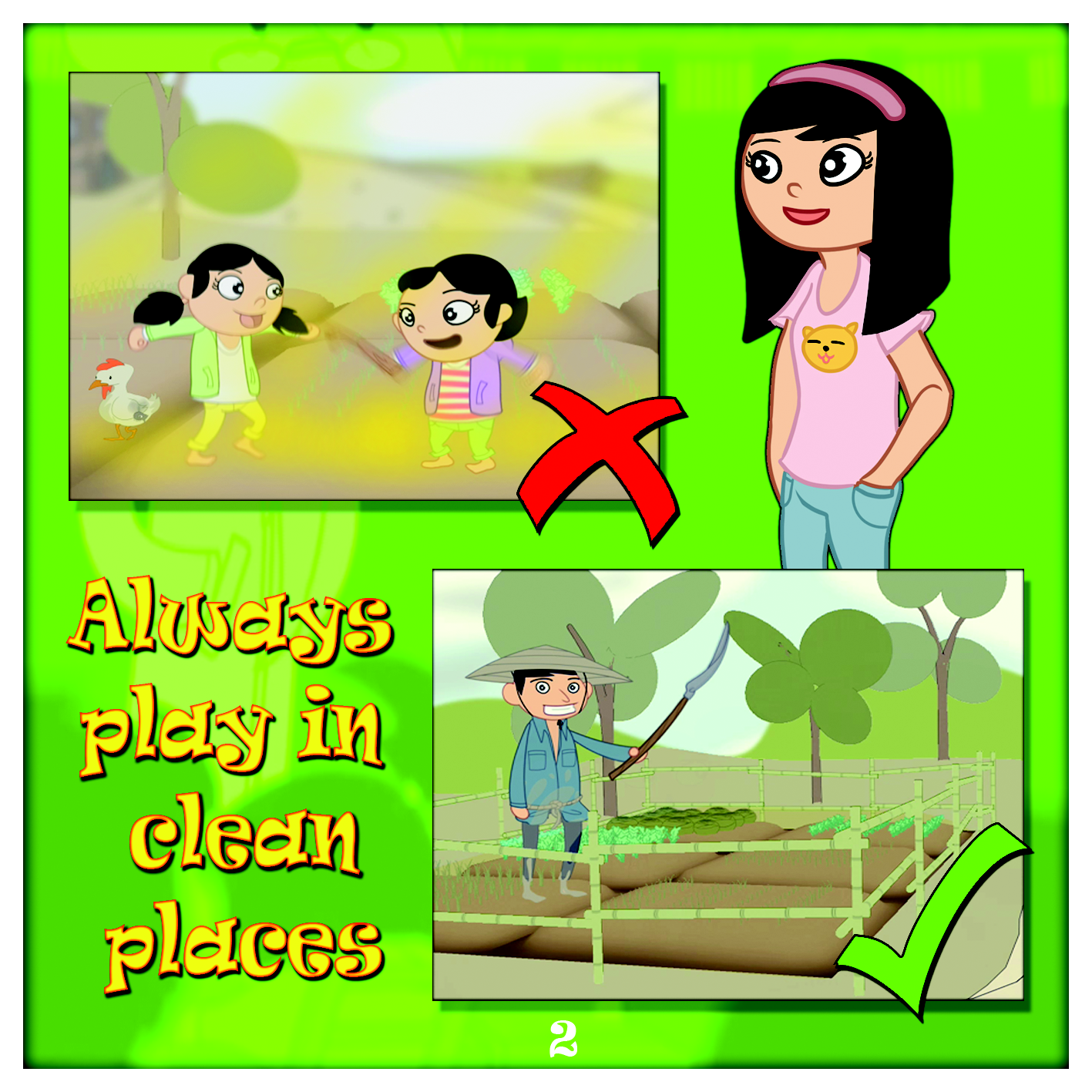


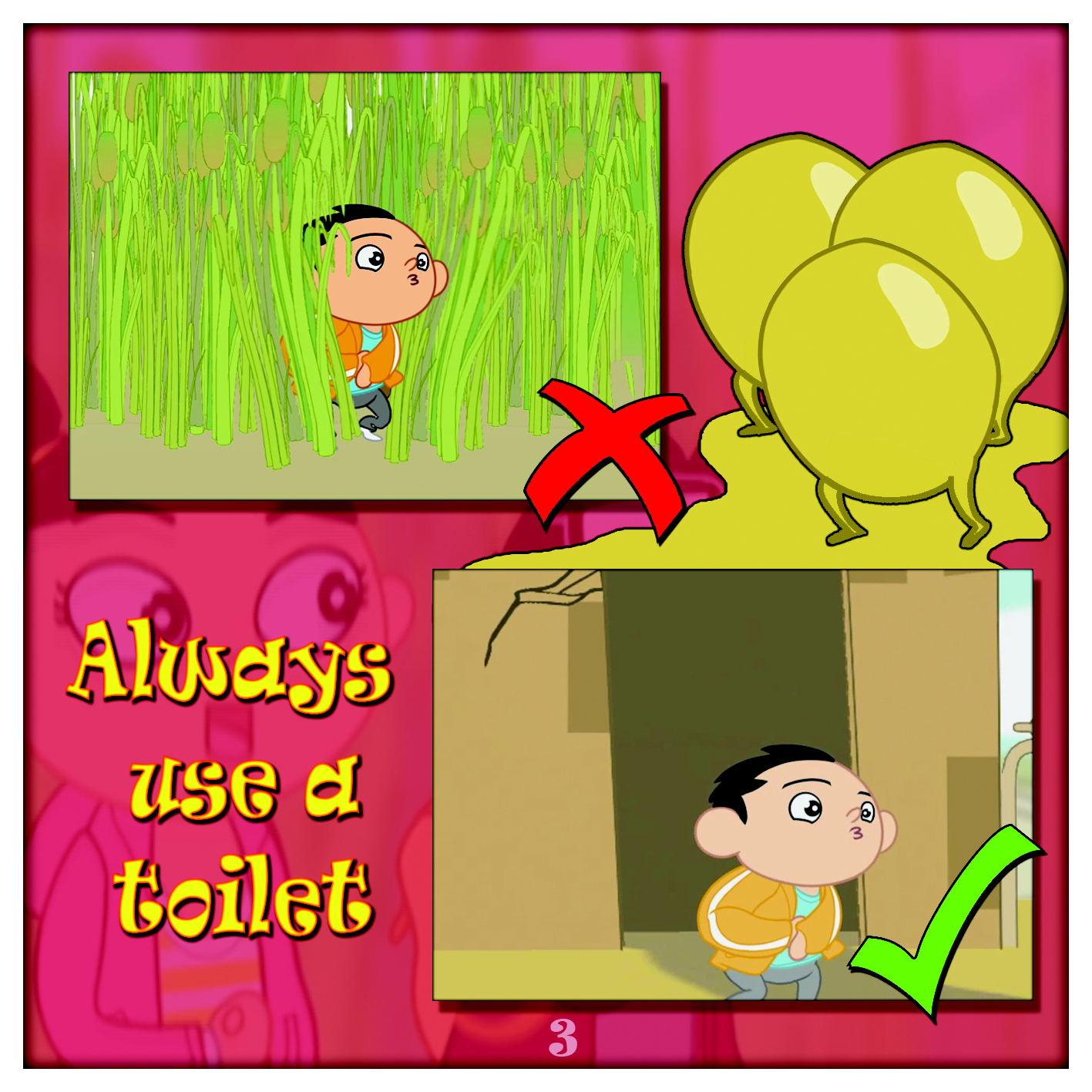

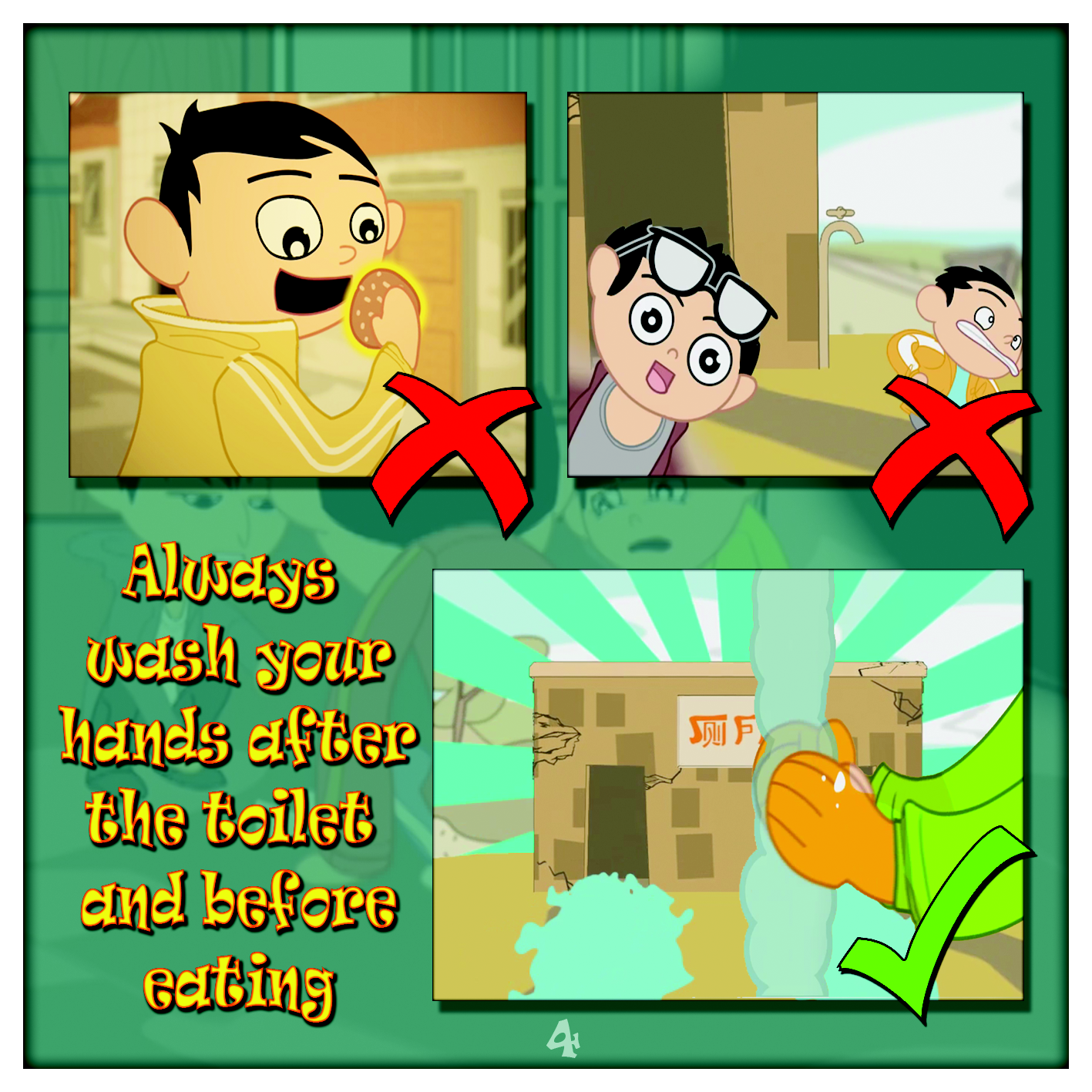

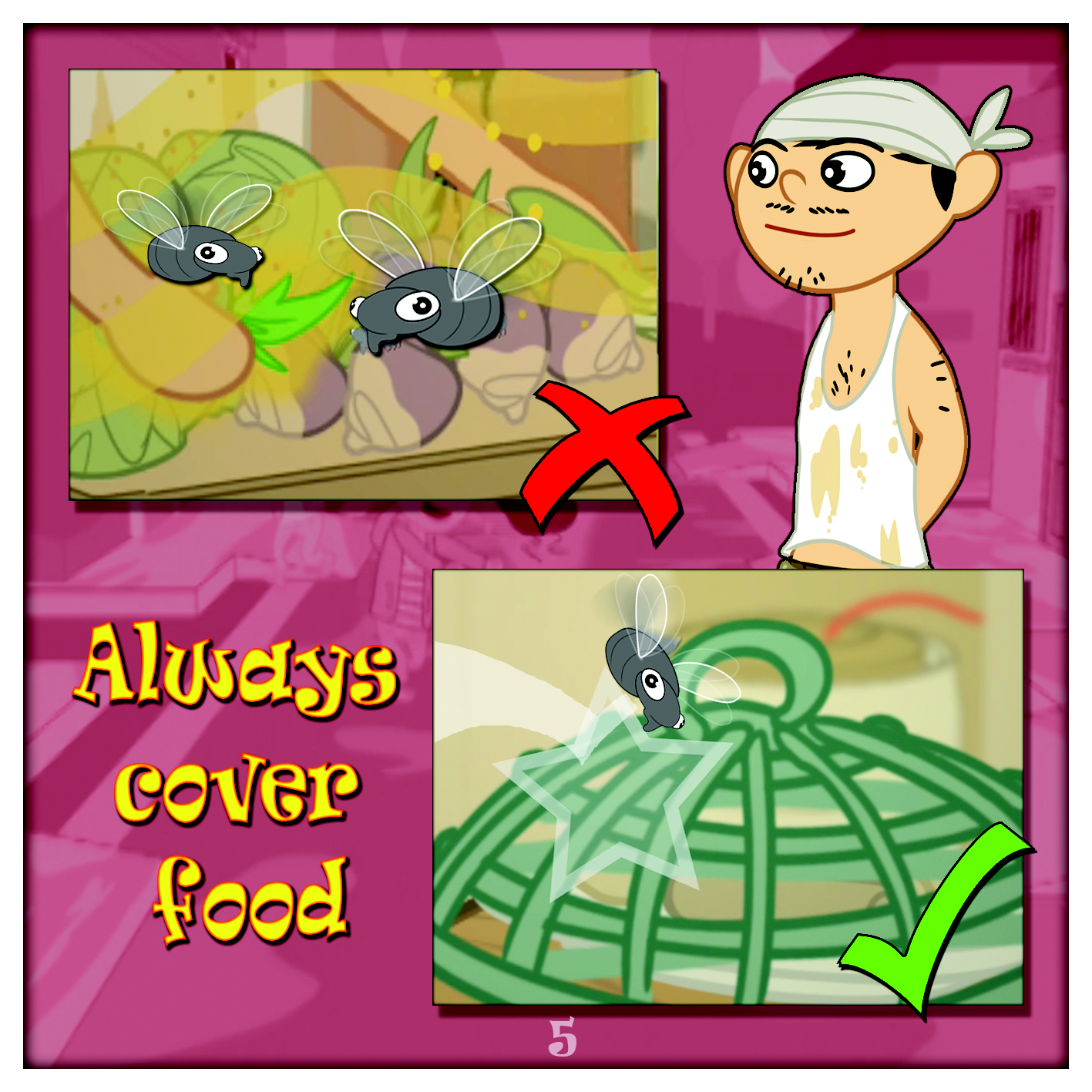

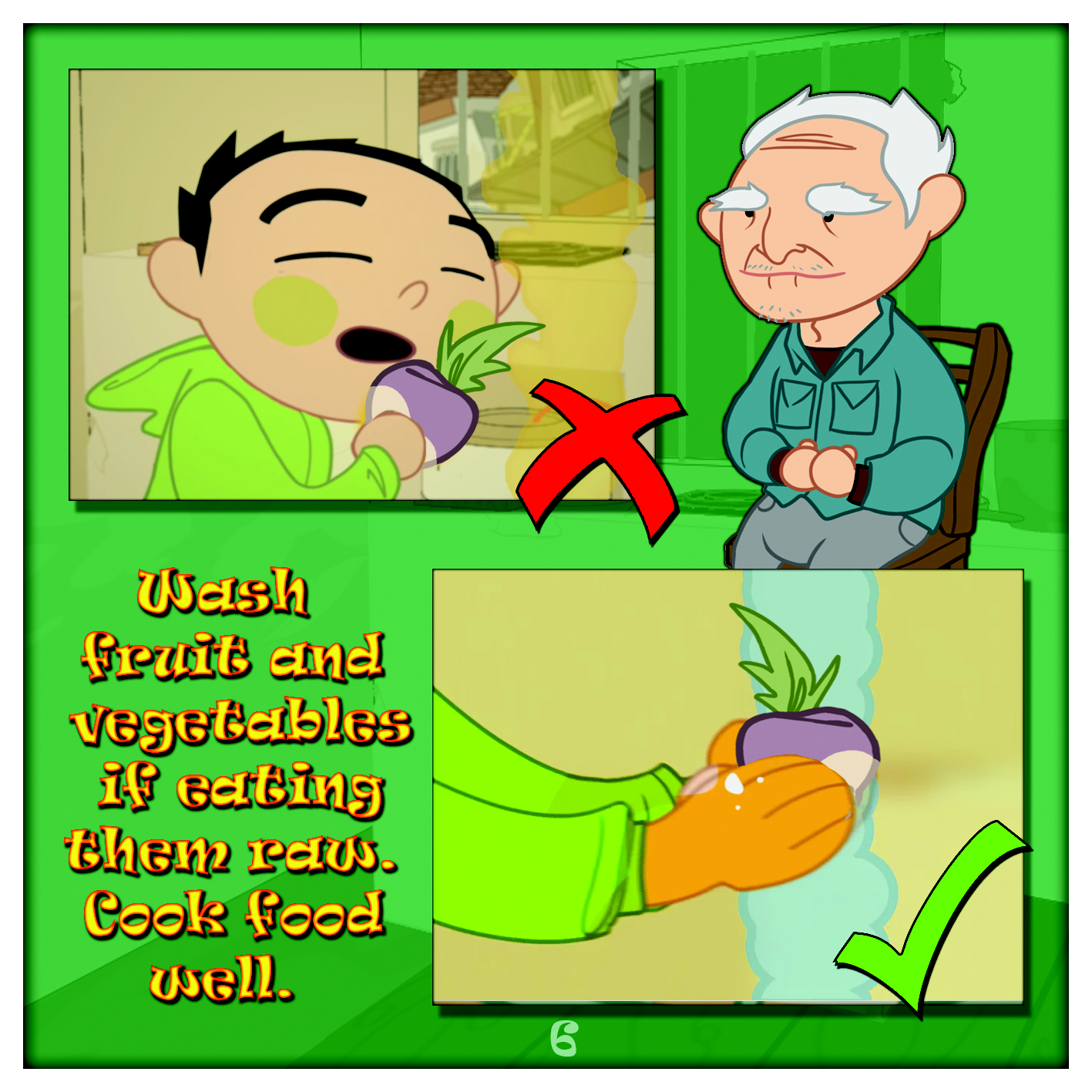

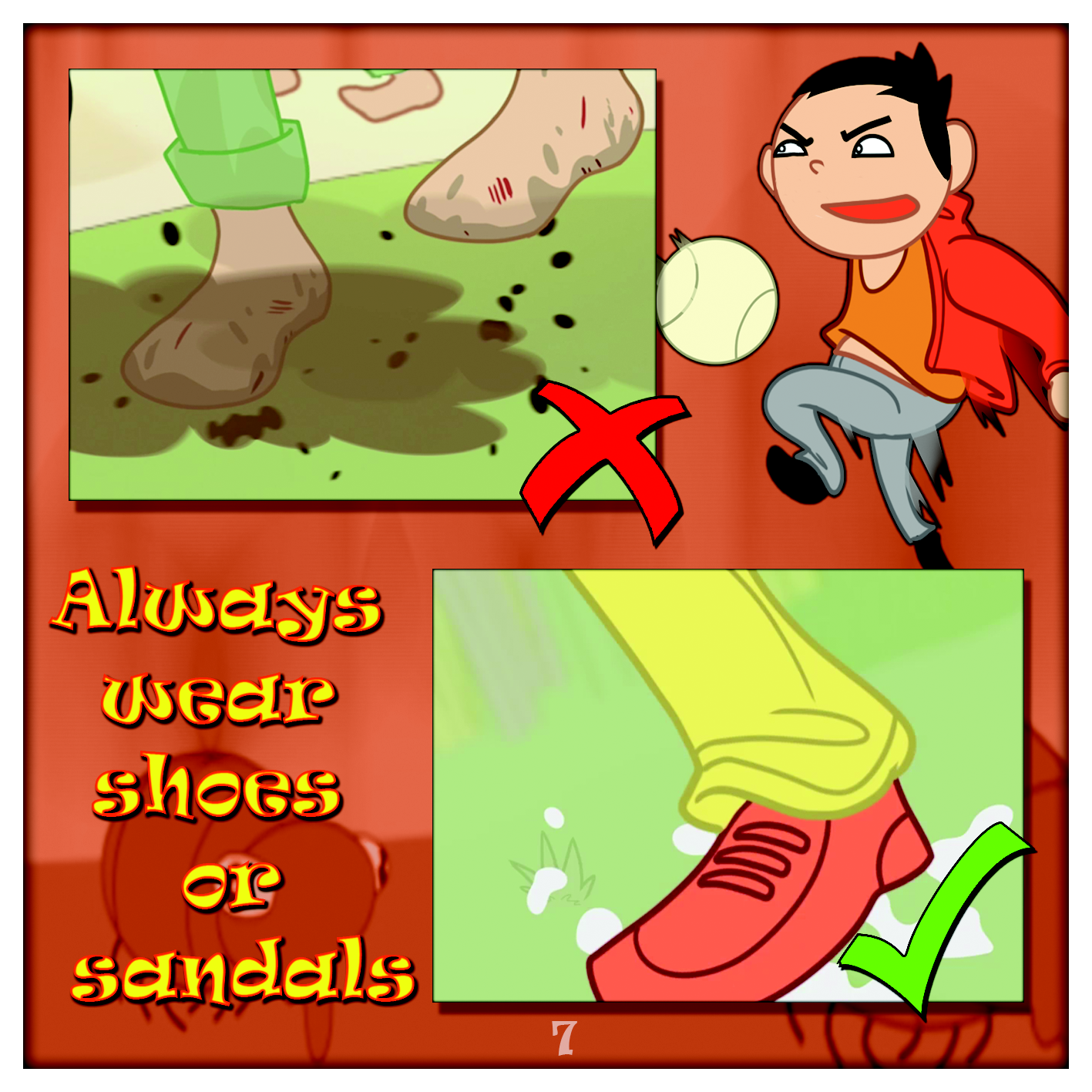

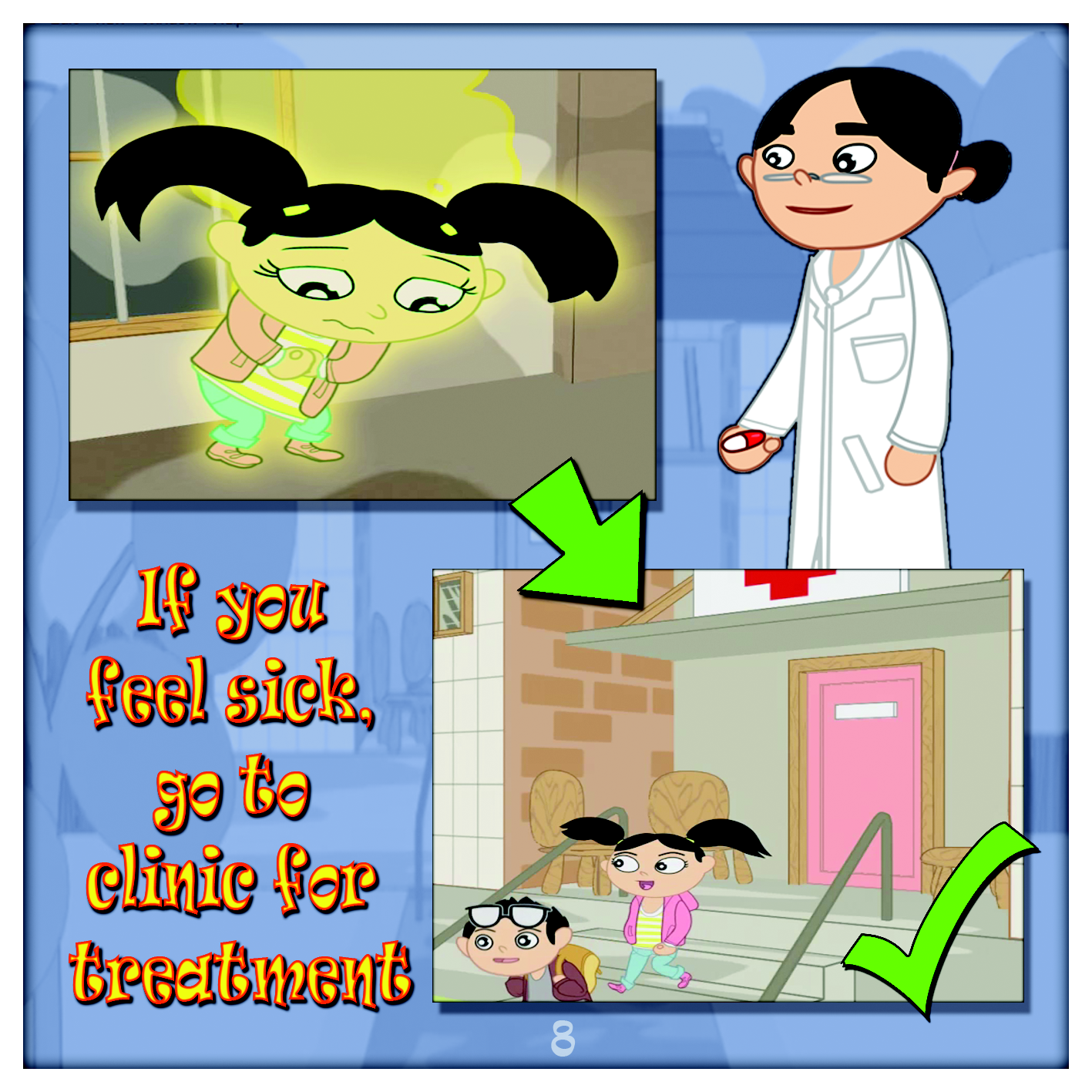

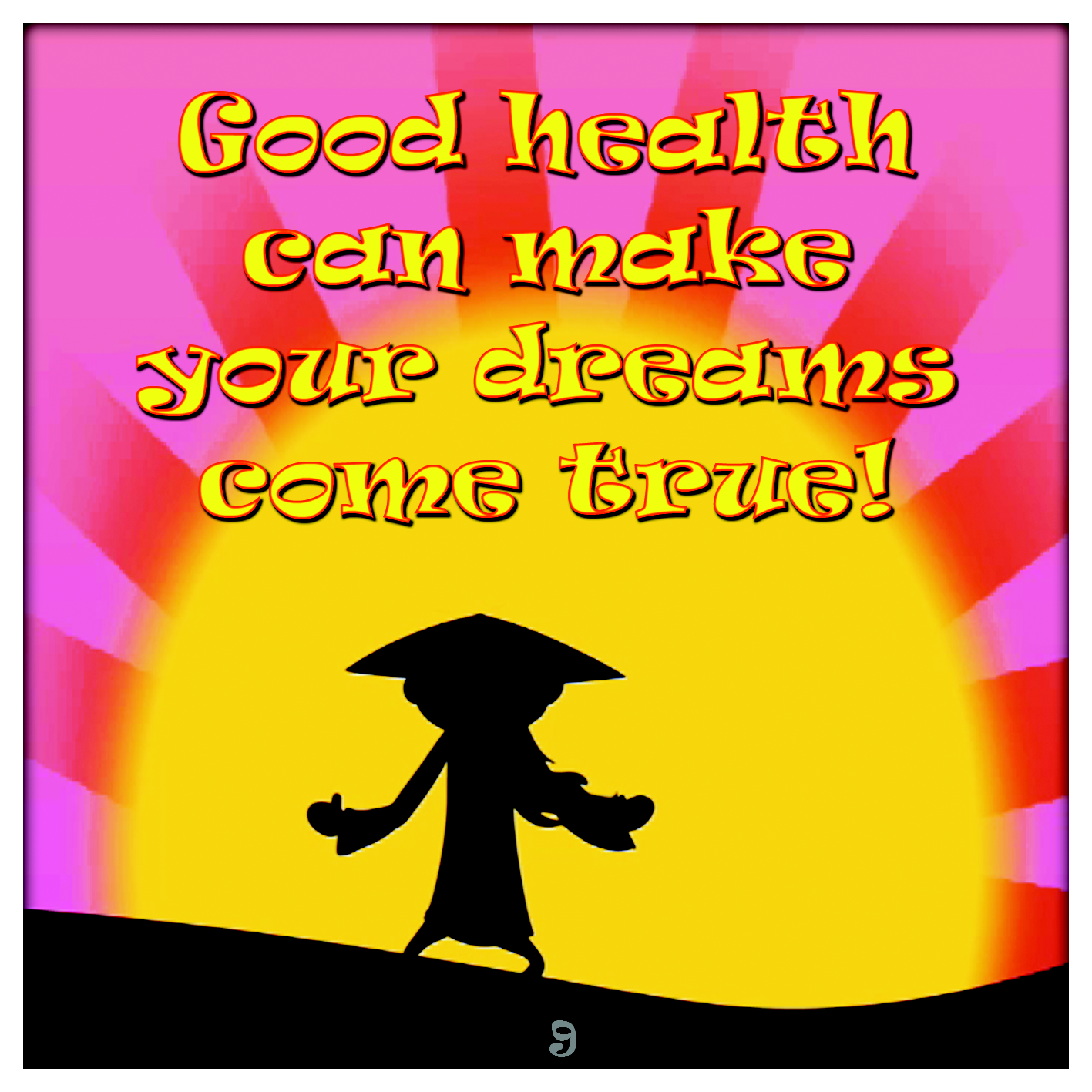

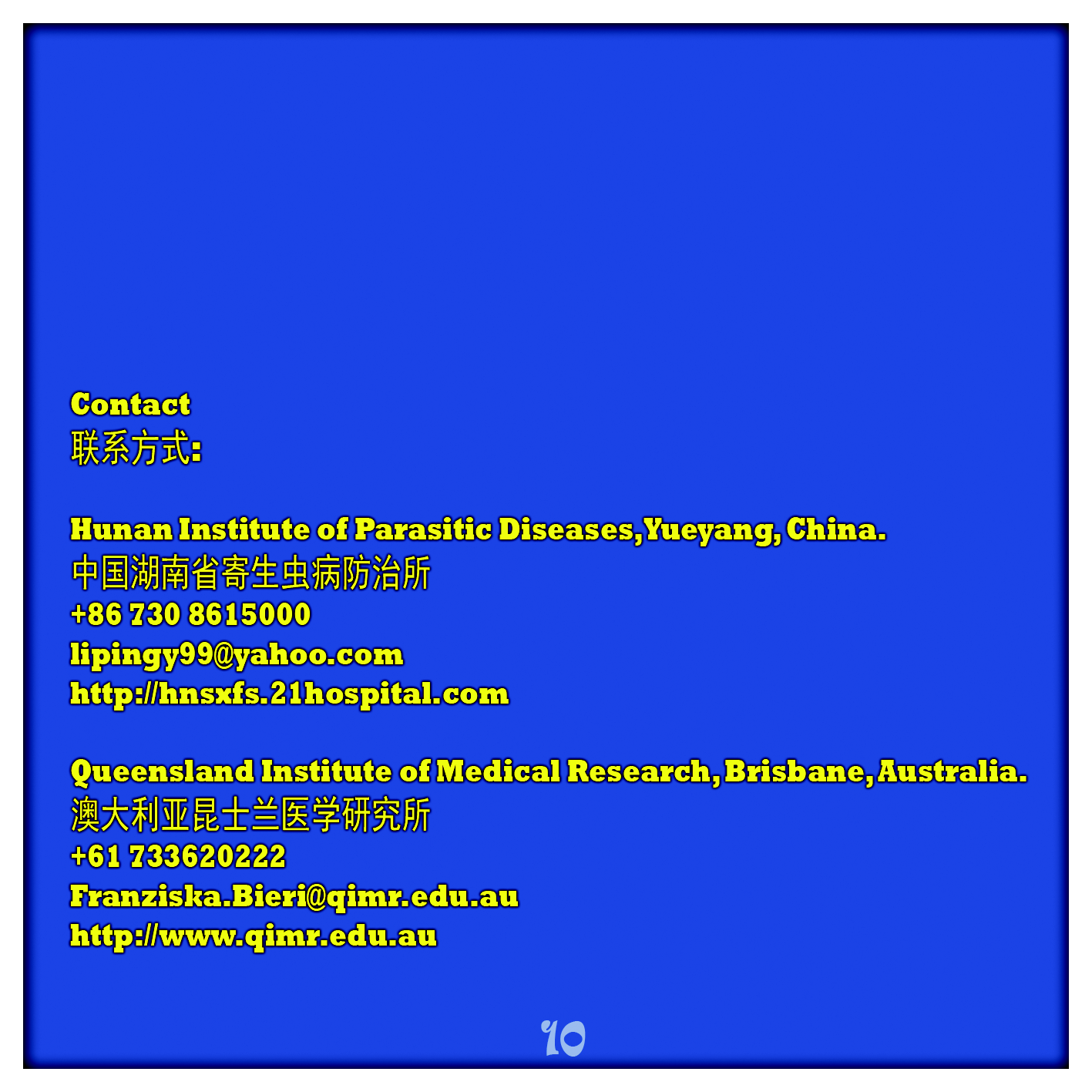

Supplement: Additional file 2 — Pamphlet with the key messages of the cartoon as a PDF. [file 2049-9957-2-29-S2.doc]
